# Post-football Gonathrosis: Injuries and Surgeries are A Risk

**DOI:** 10.7759/cureus.2953

**Published:** 2018-07-10

**Authors:** Muhammad Mannan Ali Khan, Adeel A Siddiqui, Uzair Yaqoob, Muhammad Danial Yaqub, Osama J Khan, Faizan -ul-Haq

**Affiliations:** 1 Sindh Medical College, Dow University of Health Sciences, Karachi, PAK; 2 Orthopedic Surgery, Dow University of Health Sciences, Karachi, PAK; 3 Surgery, Jinnah Postgraduate Medical Centre, Karachi, PAK; 4 Sindh Medical College, Dow University If Health Sciences, Karachi, PAK

**Keywords:** gonarthrosis, osteoarthritis, football injuries

## Abstract

Football is one of the most popular sports in the world. Many studies have shown there is a high incidence of gonarthrosis in football players. The reason for this increase is said to be injuries to the meniscus, the anterior cruciate ligament (ACL) and the resulting surgeries. The incidence is significantly increased in players with knee injuries. The knee is also the most commonly injured site in football and the most common cause of surgery in football players. Together these injuries, particularly of the ACL or meniscus and the resulting surgeries, increase the risk of developing gonarthrosis in post-football years.

## Introduction and background

Football is one of the most popular sports in the world with over 300 million players involved in it. Many studies performed show that there is a high prevalence rate (60%-80%) of osteoarthritis (OA) in footballers post-retirement [1]. Football is a very dynamic game involving physical activities like running, jumping, dribbling and tackling; however, there is no evidence that suggests any of these activities are directly linked to the development of osteoarthritis in their post-football days [2]. However, researches have generally shown that there is an increased occurrence of osteoarthritis in former elite players as compared to age-matched controls. A study published in the United Kingdom reported that knee injuries, especially of the cruciate ligament and meniscal injuries, are the cause of nearly half of all the injuries that resulted in forceful early retirement in football players during the period of 1987-1988 to 1994-1995 [3]. According to an article published in the journal named *Clinics in Sports Medicine*,* *it was suggested that the primary cause of osteoarthritis in football players is injuries to the meniscus, the anterior cruciate ligament, and the surgeries as a result of these injuries [4]. The reason for this may be the limited healing capacity of the cartilage and other intra-articular soft tissues such as anterior cruciate ligament (ACL) and meniscus that joint injuries often lead to the development of disabling osteoarthritis. This is one of the reasons why osteoarthritis is five to 12 times more common in football players as compared to the general population and is diagnosed four to five years earlier as well [5].

## Review

Osteoarthritis

Osteoarthritis is a degenerative joint disease which involves the joint cartilage and its surrounding structures. During the course of the disease, there is damage of articular cartilage, remodeling of sub-articular bone, osteophyte formation, ligament laxity, weakening of peri-articular muscles and in some cases synovial inflammation may also be seen [6]. Osteoarthritis results from a failure of chondrocytes to maintain a balance between synthesis and degradation of the extracellular matrix proteins which include two components, tissue fluid and a framework of structural molecules consisting of collagen type II, proteoglycans, and non-collagenous proteins and glycoproteins [7]. About 13% of women and 10% of men aged 60 years or older have symptomatic knee osteoarthritis. These percentages are likely to increase due to increasing age of the population and increased rate of obesity in the general population along with other risk factors which include sports participation, injury to the joint, genetic susceptibility, female gender, bone density, muscle weakness, joint laxity. Mechanical forces exerted on joints are one of the most modifiable risk factors and it can be determined using a person’s basal metabolic index. Female sex, lower education levels, obesity, and poor muscular strength are important risk factors for symptomatic disease and subsequent disability [8]. The result of these changes in the joint includes joint pain, dysfunction and ultimately in advanced stages joint contractures, muscle atrophy, and limb deformity [7]. In advanced stages the patient may also be in psychological distress and it is important to screen the patient for signs of anxiety like abnormal posture to avoid excessive pain, insomnia, and signs of depression like early morning wakening, irritability, weight loss, problems remembering things or concentrating [9]. The diagnosis of osteoarthritis is usually made on the basis of history and clinical examination. The role of radiology is usually to confirm the diagnosis and rule out other conditions [9]. Treatment options for osteoarthritis are divided into non-pharmacological and pharmacological modalities [10]. Weight reduction is one of the first steps in the management of osteoarthritis [11]. In a study published by Huang et al., it was seen that pain reduction and improvement of movement was seen in the population having OA who were undergoing weight reduction therapy [11]. Other non-pharmacological approaches include the avoidance of activities that put excessive stress on the joints. However, the most commonly used modality is rehabilitation and physical therapy [12]. Pharmacological management includes the use of acetaminophen, aspirin, and other non-steroidal anti-inflammatory drugs (NSAIDs) for the relief of pain. Glucosamine sulfate and chondroitin sulfate are used as nutritional supplements as they are used in the manufacture and repair of cartilage [13]. The surgical procedures performed include arthroscopic surgery for OA with meniscal tear, osteotomy to correct misalignment and total joint replacements. A new treatment modality that uses tissue engineering and biological therapy is called autologous chondrocyte implantation in which chondrocytes form a normal cartilage are harvested and implanted into the defected cartilage affected by OA [14]. Despite all these treatment modalities OA causes major disability in the population with an estimated 22.5 million adults from the United States (US) having difficulty walking three city blocks and an estimated 21.7 million adults from the US having difficulty climbing stairs [15].

Osteoarthritis in knee joint

Knee is the most commonly affected joint in osteoarthritis with an involvement of 41% as compared to 30% in hands and 19% in hips [16]. According to a study published by Zhang et al. Symptomatic gonarthrosis affects 13% of women and 10% of men aged 60 years and older. In a study conducted by Duncan et al. the most common pattern of radiological osteoarthritis involvement of these two joints is in combination (40%), with isolated patello-femoral joint (PFJ) involvement seen in 24% and isolated tibio-femoral joint (TFJ) involvement seen in 4% only. [17] Prolonged squatting is an important risk factor for tibio-femoral knee osteoarthritis in the elderly population [18]. Along with this obesity and meniscectomy are also stronger risk factors for TFJ disease while Heberden’s nodes and family history are more closely associated with PFJ involvement [8]. The most important exogenous risk factors for developing Secondary OA are injury related macro-trauma, repetitive micro-trauma, increased BMI and previous surgery [19]. The direction of mal-alignment of knee serves as an indication for the type of OA in the knee joint, frontal plane mal-alignment may indicate patella-femoral joint OA and tibio-femoral joint OA, valgus mal-alignment is associated mostly with lateral patella-femoral joint OA, varus mal-alignment with medial tibio-femoral joint OA with the former one i.e. lateral PFJ OA being more common of the two [20]. The sign and symptoms of moderate to severe isolated PFJ OA include dramatic swelling in the past, valgus mal-alignment, marked reduction in quadriceps strength, and pain on compression while those of TFJ joint involvement include history of previous trauma, varus mal-alignment, bony enlargement, reduced knee flexion range of motion, and fixed flexion deformity [21]. The treatment options for knee osteoarthritis are similar as discussed above, which include weight reduction, avoidance of excessive stress on knee joints, analgesics, use of supplements like proteoglycans and chondroitin sulfate, physical therapy, and rehabilitation. In severe cases, surgery is opted which includes total and partial knee replacements [10].

Osteoarthritis in football players: Cause Football, Injuries or surgery

OA has been seen to be a common occurrence in football players after their retirement from the sport. In a study conducted on 117 former top-level athletes, it was found that the prevalence of tibio-femoral and patella-femoral OA was 31% in weightlifters, 29% in football players, 14% in runners and 3% in shooters. It was also found that football players had the highest prevalence of tibio-femoral OA and weight lifters had the highest prevalence of patella-femoral OA [22]. Another study conducted by Krajnc et al. (2010) measured the prevalence of gonarthrosis in 40 former Slovenian football players and reported a 60% rate of gonarthrosis [23]. In another study conducted by Drawer et al. on 500 former players registered by the English Professional Footballers’ Association (PFA) with a response of 37% (185), he showed that around 47% respondents retired early due to injury out of which 42% were due to acute injuries and 58% were due to chronic injuries. He further showed that both of these acute (46%) and chronic (37%) injuries were most common of the knee. Out of those 185 respondents, the prevalence of medically diagnosed OA was 32%, among whom the rate of developing OA was double (51%) in the players who retired through injury in contrast to the players who didn’t retire through injury (25%) [24]. Roos et al., in their comparative study between elite football players, non-elite football players, and normal age-matched controls found that the prevalence of gonarthrosis in normal age-matched controls was 1.6%, non-elite players was 4.2% and among elite players was 15.5%. If the number of players with injuries from the non-elite group was excluded, there was no difference in the prevalence of gonarthrosis between the two groups but the prevalence was still high in the elite group [25]. Table 1 shows the prevalence of Radiographic gonarthrosis in football players in comparison to age-matched normal people. Results show that there is an increased prevalence of gonarthrosis in football players.

**Table 1:** Prevalence of Radiographic Gonarthrosis

Author	In footballers	In Normal
Petrillo S. et al. [26]	53.7%	31.9%
Paxinos O. et al. [27]	52%	33%
Chantraine A. et al.[28]	56%	-

So should football be included as a cause of OA as an occupational illness? Spahn in 2015 analyzed multiple articles to find if playing football without injuries is a cause of development of OA and found out that there is only a slight increased risk (relative risk=1.3) of gonarthrosis in players without injury [29]. In contrast in studies without differentiation between injured and non-injured knee the risk was significantly increased. (Relative risk=2.9). Gillquist et al. in their study also showed that radiographic OA of knee is significantly increased after all knee injuries [30]. So exogenous, contact related trauma and the resulting injuries are listed as the major predisposing factors for the occurrence of early OA in football players [29]. In a review article, Blagojevic et al. stated that previous knee trauma increases the risk of OA 3.86 times [31].

The incidence of knee injuries of all the football injuries is 15-19%, out of which 35%-37% are strains, 20-21% are sprains and 16-24% contusions; they constitute 58% of all the major injuries [32]. Of the ligamentous injuries, medial collateral ligament (MCL) is the most commonly injured ligament. In their study, Price et al. found that MCL injuries accounted for 85% of all the knee injuries suffered by football players [33]. ACL injury carries the highest morbidity with a successful return to play to pre-injury levels after surgery ranging from 50%-90% [34]. Janine et al. in their research performed on 217 players from eight Dutch clubs found out that the overall incidence of injuries was 6.2 per 1000 player hours with training incidence of 2.8 and match incidence of 32.8. A team sustained an average of 1.1 injuries per match. The results also showed that the most common body part injured was the knee with an incidence of 21.3% [35].

Table 2 shows the incidence of knee injuries in football players and the incidence of knee surgeries as a result of these injuries. Results show there is a high incidence of knee injuries in football players and a high number of surgeries are done as a result of these injuries.

**Table 2:** Incidence of Knee Surgeries and Injuries in Football Players

Authors	Knee injuries in football players	Knee surgeries in football players
Krajnc Z. et al.[36]	73%	43%
Musumeci G. et al. [18]	17%	52%

Soccer is one of the sports with the highest number of ACL injuries with an incidence rate of 0.15%-3.67% per person per year [37]. A study performed on elite European football showed that a high-level men’s team can expect 0.4 ACL injuries per season [38]. Tears of the lateral meniscus are more common in football than any other sports. Knee injuries are also the most common reason for surgery in football players [39]. These injuries and the resulting surgeries increase the risk of gonarthrosis in football players. Gillquist et al. showed that isolated meniscus rupture and subsequent repair, partial or total ruptures of the ACL without other major injuries increase the risk tenfold to around 15%-20% incidence of OA as compared to the age-matched, uninjured population in which the incidence is only 1%-2% [30].

Table 3 shows that there is a high prevalence of radiographic gonarthrosis in football players with ACL injuries.

**Table 3:** Prevalence of Radiographic Osteoarthritis with Anterior Cruciate Ligament Rupture

Author	Prevalence
Von Porat A. et al. [40]	78%
Lohmander LS. et al. [41]	51%

In a retrospective review, Neyret et al. analyzed 91 knees having the same operation, rim-preserving meniscectomy, with a follow up of average 27 years and found that in patients operated with intact ACL the prevalence of radiological diagnosed OA was 24% and with ruptured ACL was 77% [42]. Meniscectomy in a join with intact ligaments doubles the risk of development of gonarthrosis to 30%-40% while 50%-70% patients with complete ACL rupture and associated injuries have radiographic changes of OA after 15-20 years [30]. A recent study has showed that all footballers under-going revision ACL surgery had OA when they were examined 37 months after reoperation [43]. Smith et al., in their research, showed that the incidence of gonarthrosis was only 4% in those American football players without any previous knee surgery, 11% in those with history of meniscus repair and 24% of those with a history of ACL reconstruction. It was also noticed that in knees with previous ACL reconstruction, the rate of OA in the tibio-femoral compartment doubled in those patients who have had previous meniscal surgery [44]. The cost of ACL injuries is estimated to be US $4 billion for surgical treatment alone and US$ 7.6 billion annually when treated with ACL reconstruction and US $17.7 billion when treated with rehabilitation. Even after so much effort, 59%-70% will develop radiographic OA, 16%-19% will have symptomatic gonarthrosis in the course of their lifetime, and 13-15% will need total knee replacements [45].

## Conclusions

From the above observations, it can be concluded that football is one of the most common sports being played throughout the world at the present time. Playing football in itself increases the risk of developing gonarthrosis only slightly but is still higher than the age-matched normal population. The reason for this is said to be the repetitive micro-trauma suffered by the joint due to the vigorous training and playing hours. The risk for developing gonarthrosis is significantly increased due to injuries particularly the ACL injury or injury to the meniscus. Football is the sport with the highest number of ACL injuries. Surgeries, particularly for ACL reconstruction or meniscectomy, performed further increases the risk of developing gonarthrosis in the post-football life.
